# Mukonal exerts anticancer effects on the human breast cancer cells by inducing autophagy and apoptosis and inhibits the tumor growth in vivo

**DOI:** 10.1186/s13568-020-01074-8

**Published:** 2020-08-18

**Authors:** Wenbiao Wang, Zhengui Zhou, Xiaohong Zhou, Limin Chen, Shuang Bie, Zhengjun Jing

**Affiliations:** Department of Thyroid Breast Surgery, Gong An County People’s Hospital, Gong An, Jingzhou, 434300 Hubei China

**Keywords:** Breast cancer, Alkaloids, Carbazole alkaloids, Mukonal, Autophagy, Apoptosis

## Abstract

Mukonal is an active member of carbazole alkaloids isolated from *Murraya koenigii.* It has been shown to possess remarkable biological and pharmacological activities including anticancer activity. Therefore, the aim of current investigation was to explore anti-breast cancer activity of mukonal and to explore the underlying mechanism. Results indicate that mukonal has potential to induce antiproliferative effects against MDA-MB-231 and SK-BR-3 cells with an IC_50_ of 7.5 µM. No significant toxicity of mukonal was observed in case of normal breast cells. The antiproliferative effects of mukonal were found to proceed via apoptosis, which was further supported by increased cleavage of PARP and caspase-3 and reduced expression of Bcl-2. Mukonal induced autophagic cells death in breast cancer cells as was evidenced by formation of autophagosomes and enhanced expressions of Beclin-1, LC3B-I and LC3B-II proteins. In vivo examination of anti-breast cancer property of mukonal indicated that it could potentially reduce tumor weight and volume in xenografted mice models. In conclusion, mukonal has a remarkable potential of inhibiting breast cancer via induction of apoptosis and autophagy. Mukonal also inhibited xenografted tumors models in a dose-dependent manner. Therefore, mukonal may prove lead molecule in breast cancer drug discovery and treatment provided further investigations are recommended.

## Introduction

Breast cancer is the most frequent and devastating disease prevailing in females worldwide. Alone in the year of 2015, more than 0.57 million deaths were recorded globally due to this lethal malignancy in women (Sun et al. 2017). Every year, approximately 25% of all cancers (over 1.5 million) diagnosed in women are breast cancer. United States registered over 30% of all cancers in women were due breast cancer in the year of 2017 (Siegel et al. 2017). Metastatic nature of breast cancer leads to easy transfer of disease to distant places in the body wherein it develops individually like brain, lung, liver and bones. Early detection of the disease may lead with better overall survival chances and prognosis. Screening of breast cancer is widely performed by mammography which has been proved fruitful in lowering the mortality rate effectually. Several risk factors have been linked to enhance the possibility of breast cancer development like that of genetic mutations, family history, estrogen levels, aging, sex and poor lifestyle (Majeed et al. 2014). Primary development of breast cancer takes place from ductal hyperproliferation and then maturing into metastatic or benign tumors. Thus far, significant advances have been achieved in theoretical and clinical investigations of breast cancer. The current strategies for effective management of breast cancer incorporate biological-prevention, chemoprevention and screening (Smith and Henderson 1996). Besides recent advances and effective management, breast cancer remains major cause of cancer related deaths in females of age 20–60 years. Therefore, there is a pressing need for novel and capable chemopreventive drugs that can assists us with better results in overcoming this disastrous malignancy. Since time immemorial, natural products have revealed health promoting effects in human beings. Natural products are mostly the secondary metabolites synthesized by the plants to survive and adapt to harsh environmental conditions (Williams et al. 1989). Alkaloids are a major class of naturally occurring secondary metabolites in plants with huge medicinal and biological properties including anti-diabetic, analgesic, anti-inflammatory, anti-hypertensive, anti-malarial and anticancer. Mukonal (molecule is an active member of carbazole alkaloids found in *Murraya koenigii* (Bhattacharyya and Chakraborty 1984). This molecule has shown significant antioxidant, antimicrobial and anticancer activity (Samanta et al. 2017). Mukonal has been reported to show anticancer activities against different human cancer cell lines in vitro including laryngeal AMC-HN-8 cancer cells and nasopharyngeal CNE-1 carcinoma cells. It has shown a remarkable potential of autophagy initiation, apoptosis induction, modification of mitochondrial membrane potential, cell cycle arrest, blocking of MEK/ERK and PI3K/AKT signalling pathways (Li et al. 2018; Guo et al. 2019). Therefore, current investigation was designed to unveil the anti-breast cancer potential of mukonal along with its effects of autophagy and apoptosis induction.

## Materials and methods

### Reagents, chemicals and cell cultures

Mukonal with 98% of purity by HPLC and other chemicals were bought from Sigma-Aldrich (Darmstadt, Germany) unless otherwise mentioned. Breast cancer cell lines MDA-MB-231, CAMA-1, MDA-MB-436 and SK-BR-3 and normal breast cell line MB-157 were procured from Type Culture Collection of Chinese Academy of Sciences; Shanghai, China. All cell cultures were grown in RPMI-1640 media maintaining 10% fetal bovine serum (Thermo Fisher Scientific, Inc., Waltham, United States) and penicillin G (100 U/ml) and streptomycin (100 µg/ml) as suitable antibiotics. All-inclusive cell cultures were placed and maintained within a humid environment of 5% CO_2_ concentration and a temperature of 37 °C.

### Determination of cellular proliferation

The cellular proliferation of (MDA-MB-231, CAMA-1, MDA-MB-436 and SK-BR-3) and a normal breast MB157 cell line were estimated after mukonal exposure by 3-(4,5-dimethylthiazol-2-yl)-2,5-diphenyl tetrazolium bromide (MTT) assay. In brief, both cancer and non-cancer cells were placed with a concentration of 1.5 × 10^4^ cells per well of 96-well plates and precultured for 24 h in a humid environment of 5% CO_2_ concentration and a temperature of 37 °C. Thereafter, each well plate was supplemented with different mukonal doses viz. 0, 2.5, 5, 10, 20, 40, 80, 160, 320 and 640 µM, and left untouched on incubation for 48 h. Mukonal treated cancer and non-cancer breast cells were washed twice with phosphate buffered saline (PBS) prior to staining with 20 µl of MTT stock solution of concentration 5 mg/ml for 3 h. The formazan crystals then evolved were dissolved with dimethyl sulphoxide followed by colorimetric analysis. Finally, absorbance was taken at 540 nm to determine the optical density using microplate spectrophotometer (Bio‑Rad Laboratories, Inc., Hercules, United States). Experiments for individual mukonal concentrations were repeated thrice.

### Colony formation assay

MDA-MB-231 and SK-BR-3 were plated onto 6-well plates with 400 cells per well. After 24 h of preculturing the cells, treatment with variant mukonal doses of 0, 5, 10 and 20 µM was instigated. Thereafter, cancer breast cells were left on incubation for 10 days devoid of any physical and chemical disturbance. After 10 days of incubation cell colonies were stained with crystal violet. Finally, MDA-MB-231 and SK-BR-3 cell colonies were totaled under a light microscope (OLYMPUS, Japan) and only colonies with > 50 cells were considered for counting.

### Annexin V/PI staining assay

Annexin V/PI dual staining assay was performed to monitor and quantify apoptosis in mukonal treated cancer MDA-MB-231 and SK-BR-3 breast cells. These cancer cells were placed onto 6-well plates and subjected to variant mukonal doses viz. 0, 3.75, 7.5 and 15 µM for 48 h. After that cells were washed in PBS, fixed in 10% of formaldehyde and yet again washed in PBS. Finally, these cells were stained with annexin V/PI dual staining solution and eventually analyzed through flow cytometry.

### Transmission electron microscopy

Mukonal treated MDA-MB-231 and SK-BR-3 cells at variant doses (0, 3.75, 7.5 and 15 µM) were subjected to electron microscopy for autophagy assessment. Mukonal treated cancer cells were fixed in the solution of 4% glutaraldehyde bearing 0.05 m of sodium cacodylate. Afterwards, post fixation of treated cells was carried out by 1.5% of osmium tetroxide followed by moisture removal using alcohol. Then cells were prepared for implantation over Epon 812 for sectioning and finally investigated under Zeiss CEM 902 electron microscope (Oberkochen, Germany).

### Assessment of protein expressions by western blotting

The expressions levels of apoptosis and autophagy allied proteins were evaluated by western blotting. Mukonal treated (3.75, 7.5 and 15 µM) and untreated (controls) tumerous breast cells (MDA-MB-231 and SK-BR-3) were lysed with RIPA buffer for protein collection. Each lysate was subjected to bicinchoninic acid assay for quantification of proteins and 30 µg of proteins from each sample were run on 10% SDS-PAGE. Thereafter, proteins were transferred to PVDF membranes and these membranes were blocked with non-fat milk at room temperature for 1.5 h. blocked membranes were subjected to suitable primary antibodies of 1:1000 dilution anti-caspase-3, anti-PARP, anti-BAX, anti-Bcl-2, anti-LC3B-I, anti-LC3B-II and anti-Beclin-1 (Santa Cruz Biotechnology, Inc., Dallas, United States) overnight at 4 °C. Afterwards, secondary antibody treatment was instigated with 1:1,000 dilutions of horse radish peroxidase‑conjugated anti‑rabbit secondary antibody (Santa Cruz Biotechnology, Inc., Dallas, United States) for 1 h at room temperature. Finally, the protein bands were visualized using ECL Advanced Western Blot Detection kit (GE Healthcare Life Sciences, Sweden).

### Xenograft study

Xenograft studies were carried out by strictly obeying the ethical guidelines permitted by animal ethics committee Gong An County People’s Hospital, Gong An, Jingzhou, Hubei, China. Immunodeficient nude mice of 6-weeks weighing 25–30 g were placed in sterile steel cages. These cages were placed in an environment of 12 h cycle of light:dark, relative humidity of 50% and a moderate temperature of 22 °C. These mice were given subcutaneous injections of SK-BR-3 cells (4 × 10^5^) on their left flank. As the tumor became superficially apparent, treatment with mukonal was instigated by intraperitoneal insertion with 0.1% of DMS, dissolved mukonal and diluted normal saline at doses of 10, 20 and 40 mg/kg body weight. This treatment procedure was repeated each second of a week and only 0.1% of DMSO was injected to the control mice. The tumor volume and weight was monitored after every week and the procedure lasted for 6 weeks. The mice were then sacrificed for this noble cause by inhaling of deep anesthesia with isoflurane. The tumor volume was determined using the following formula: V= (W × W × L)/2; where ‘V’ is the volume, ‘W’ is the width and ‘L’ is the length of the tumor. The study was approved by the animal ethics committee of Gong An County People’s Hospital, Gong An, Jingzhou, Hubei, China, 434300 under approval number 721/GACPH/02/2019.

### Statistical analysis

Statistical analysis of data, collected by execution of each individual experiment in triplicates, was performed via analysis of variance (ANOVA), and followed by Tukey’s post-hoc test. Entire data were represented as mean ± SE (standard error) considering *p* < 0.05 as statistically significant.

## Results

### Mukonal inhibited proliferation of breast cancer cells

**Fig. 1:**
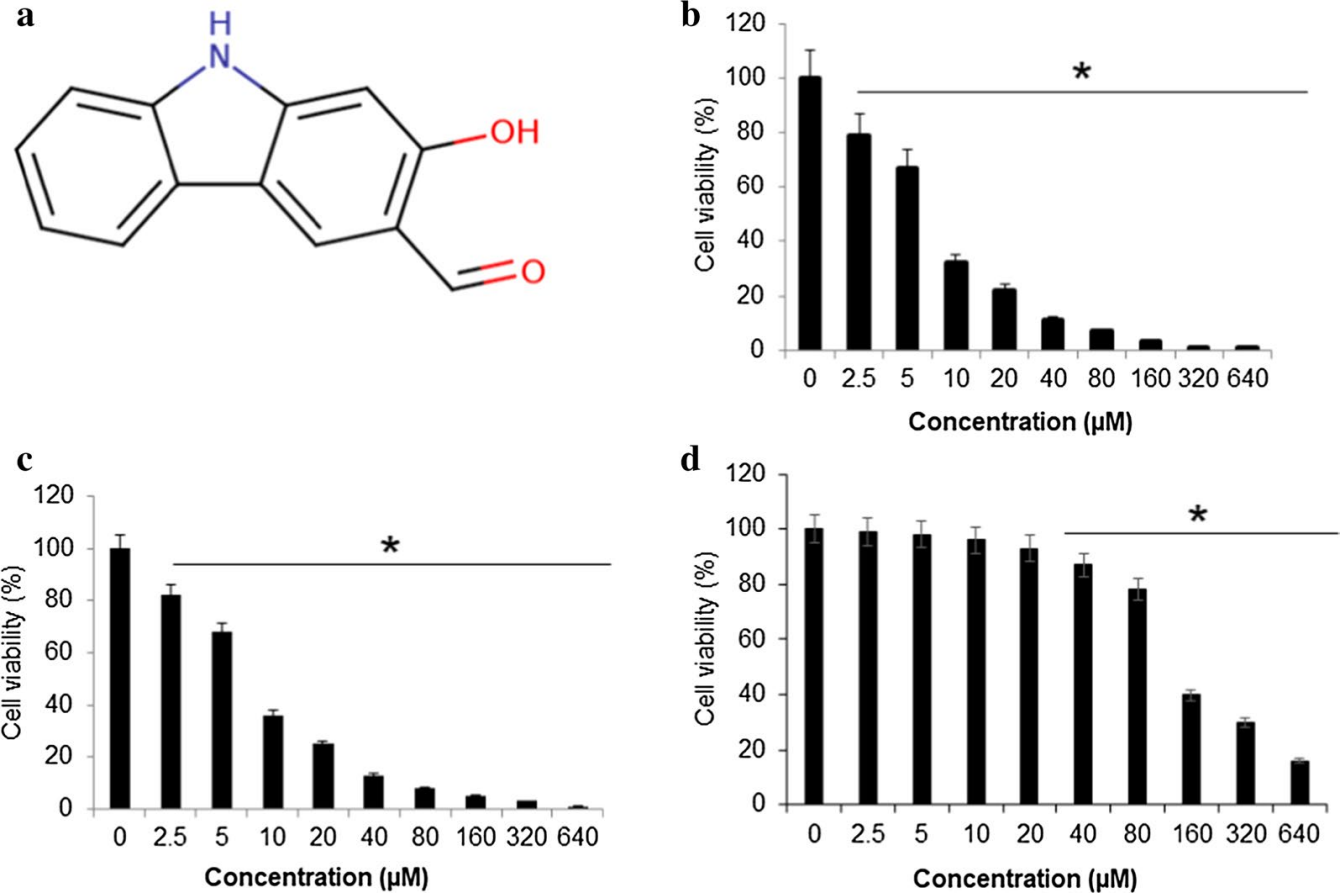
Mukonal inhibits the growth of breast cancer cells.** a** Chemical structure of mukonal molecule. **b** The viability of SK-BR-3 breast tumor cells after being subjected varying mukonal concentration as indicated. SK-BR-3 cells were exposed to mukonal for 48 h and then subsequently stained with MTT solution for calorimetric analysis. **c** The viability of MDA-MB-231 breast tumor cells after being subjected varying mukonal concentration as indicated. **d** The viability of normal MB-157 breast cells after being subjected varying mukonal concentration as indicated. All the experiments were executed three times and data was shown as mean ± SE (standard error). The p value of < 0.05 was taken as a statistical significant figure

**Fig. 2:**
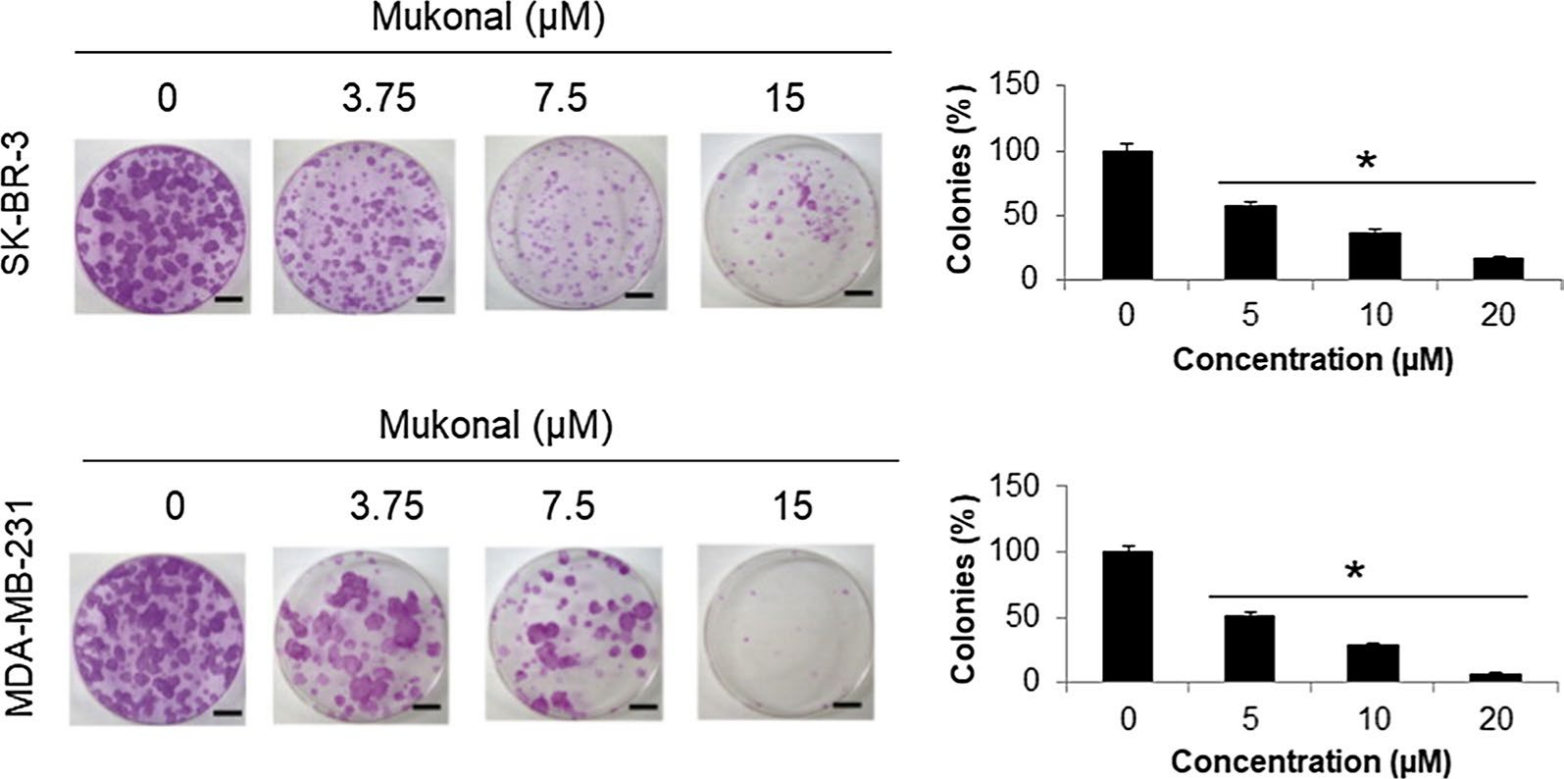
Clonogenic analysis of MDA-MB-231 and SK-BR-3 cells after being exposed to indicated mukonal doses. MDA-MB-231 and SK-BR-3 breast tumor cell lines were treated with mukonal, left untouched for 10 days and stained with crystal violet to calculate the number of colonies generated provided considering colonies with > 50 number of cells for calculation. All the experiments were executed three times and data was shown as mean ± SE (standard error). The p value of < 0.05 was taken as a statistical significant figure

The proliferation rate of four different cancer breast cell lines (MDA-MB-231, CAMA-1, MDA-MB-436 and SK-BR-3) and a normal breast MB157 cell line was determined using MTT assay after treatment with variant Mukonal 0-640 µM). Results revealed that mukonal significantly retarded the proliferation of these cancer breast cells with an IC_50_ value ranging from 7.5 µM to 25 µM (Table 1). Higher efficiency with lower IC_50_ value was recorded in case of MDA-MB-231 and SK-BR-3 cells (Fig. 1a). Therefore, rest of the studies was carried on these two cell lines. The viability in case of MDA-MB-231 cells reduced from 100% to almost 2% (Fig. 1b) and that of SK-BR-3 cells from 100% to nearly 0% with enhanced doses of mukonal from 0 to 640 µM (Fig. 1c). The mukonal induced no significant cytotoxicity against normal MB-157 breast cells and a high IC_50_ of 110 µM was obtained (Fig. 1d). Enhanced cytotoxicity was observed in case of normal MB-157 cells at higher mukonal doses. Clonogenic assay was used to monitor the impact of mukonal treatment over colony generation propensity of MDA-MB-231 and SK-BR-3 cells. On treatment of MDA-MB-231 and SK-BR-3 cells with mukonal, remarkable suppression of their colonies was observed in comparison to controls. The numbers of MDA-MB-231 colonies left over were nearly 15% and those of SK-BR-3 cells were nearly 5% after 10 days long treatment at 20 µM of mukonal concentration (Fig. 2). Therefore, mukonal induced remarkable toxicity against MDA-MB-231 and SK-BR-3 cells in comparison to MB-157 cells, which indicates specificity in anticancer activity of mukonal.

**Table 1:** Effects of Mukonal on the viability of breast cancer cells as depicted by MTT assay and presented as IC_50_

S. no	Breast cancer cell lines	IC_50_ (µM)
1	MDA-MB-231	7.5
2	CAMA-1	25
3	MDA-MB-436	25
4	SK-BR-3	7.5
5	MB-157	110

The experiments were performed in triplicate

### Mukonal induced apoptosis in breast cancer cells

**Fig. 3:**
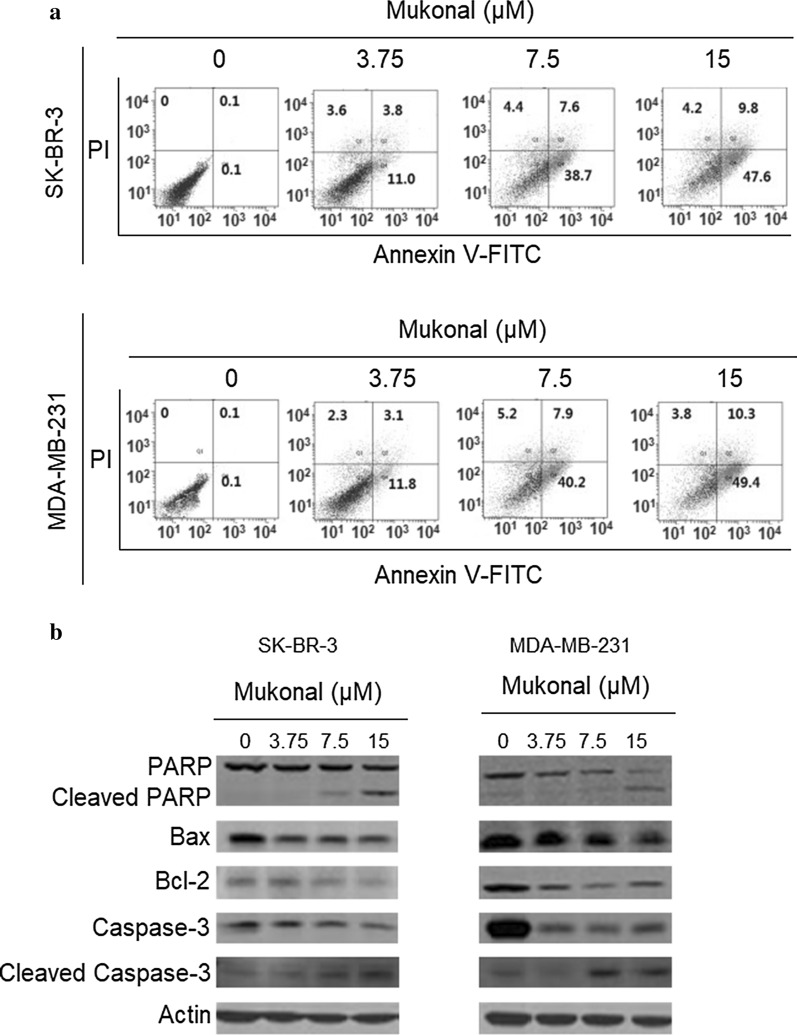
**a** Flow cytometric analysis of mukonal treated SK-BR-3 cells. SK-BR-3 cells were cultured in 6-well plates and then subjected to indicated mukonal doses. Afterwards, staining with annexin V/PI was performed to analyze apoptosis flow cytometrically. **b** Flow cytometric analysis of mukonal treated MDA-MB-231 cells. MDA-MB-231 cells were cultured in 6-well plates and then subjected to indicated mukonal doses. Afterwards, staining with annexin V/PI was performed to analyze apoptosis flow cytometrically. **c** Western blotting analysis was performed to assess the activity of apoptosis allied proteins in SK-BR-3 cells. Results indicated that mukonal enhanced activity of proapoptosis proteins wherein blocking of antiapoptotic protein expression. **d** Western blotting analysis was performed to assess the activity of apoptosis allied proteins in MDA-MB-231 cells. Results indicating that mukonal enhanced activity of proapoptosis proteins in MDA-MB-231 cells wherein blocking of antiapoptotic protein expression. All the experiments were executed three times and data was shown as mean ± SE (standard error). The p value of < 0.05 was taken as a statistical significant figure

The annexin V/PI assay was employed to determine the percentage of apoptosis in MDA-MB-231 and SK-BR-3 cells. Results indicated that mukonal could potentially exhibit proapoptotic effects against both MDA-MB-231 as well as SK-BR-3 cells in a dose-reliant fashion. In comparison to control group the number of early apoptotic, late apoptotic and necrotic cell percentage enhanced significantly in treated groups. The percentage of apoptotic cells was raised to about 61.6% in case of treated SK-BR-3 cells (Fig. 3a). The apoptotic cell percentage of MDA-MB-231 cells at controls was 0.1% but it enhanced to nearly 63.5% at 15 µM of mukonal concentration (Fig. 3b). Further, western blotting analysis indicated that Mukonal enhanced the cleavage of caspase-3 and PARP levels. Moreover, the expression of Bcl-2 decreased and Bax increased with increase in the dosage of mukonal in case of SK-BR-3 cells (Fig. 3c). The expressions of cleaved caspase-3 and cleaved PARP enhanced significantly and Bcl-2, PARP and Bax decreased with enhancing doses of mukonal in case of MDA-MB-231 cells (Fig. 3d). Therefore, the results from annexin V/PI staining and western blotting analysis indicated that antiproliferative effects of mukonal could be mediated via its apoptosis inducing propensity.

### Mukonal induced autophagy in breast cancer cells

**Fig. 4:**
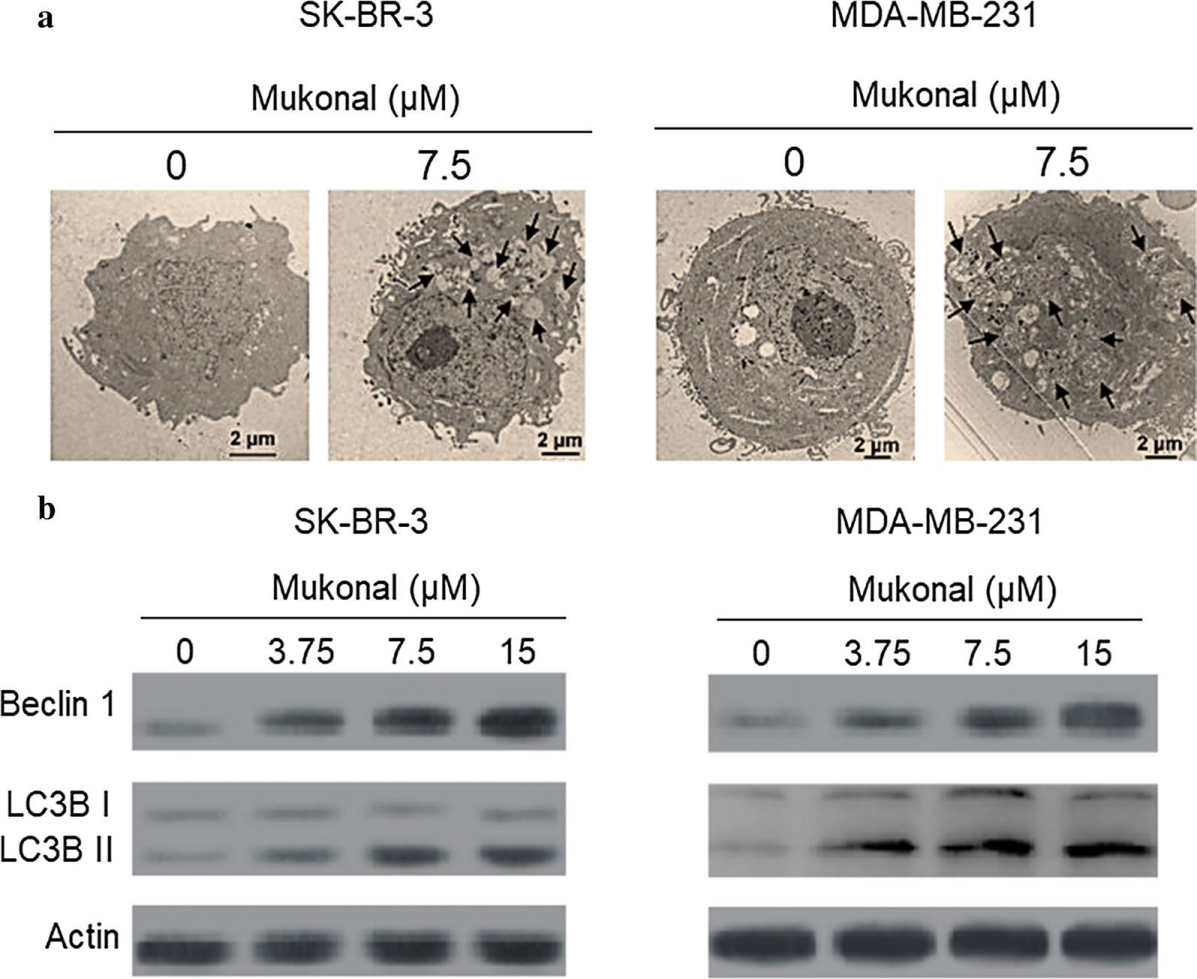
**a** TEM analysis of SK-BR-3 cells after being exposed to indicated doses of mukonal. Results revealed formation of autophagic vesicles/autophagosomes in treated groups as compared to control group. Autophagosomes have been shown by arrows in the picture. **b** TEM analysis of MDA-MB-231 cells after being exposed to indicated doses of mukonal. Results revealed formation of autophagic vesicles/autophagosomes in treated groups as compared to control group. Autophagosomes have been shown by arrows in the picture. **c** Western blotting results of SK-BR-3 cells after being exposed to mukonal at indicated doses. In treated groups enhanced activity of Beclin-1, LC3B-I and LC3B-II was observed with increasing concentrations of mukonal. **d** Western blotting results of MDA-MB-231 cells after being exposed to mukonal at indicated doses. In treated groups enhanced activity of Beclin-1, LC3B-I and LC3B-II was observed with increasing concentrations of mukonal

The impact of mukonal exposure on cellular autophagy in MDA-MB-231 and SK-BR-3 cells was analyzed through transmission electron microscopy and western blotting assay. Results indicated that mukonal potentially induce proautophagic effects in both of these cancer breast cell lines. In comparison to control groups, autophagosomes are clearly visible in both mukonal treated SK-BR-3 (Fig. 4a) and MDA-MB-231 (Fig. 4b) cells. After exposure to variant doses (0–15 µM) of mukonal, MDA-MB-231 and SK-BR-3 cell lines showed enhanced expression levels of Beclin-1, LC3B-I and LC3B-II proteins. Which indicated molecular features of autophagic cell death in mukonal treated MDA-MB-231 and SK-BR-3 cancer cells (Fig. 4c, d). Therefore, it may be concluded that mukonal exhibits antiproliferative effects mediated via its autophagy inducing potential.

### In vivo inhibition of tumor growth by mukonal

**Fig. 5:**
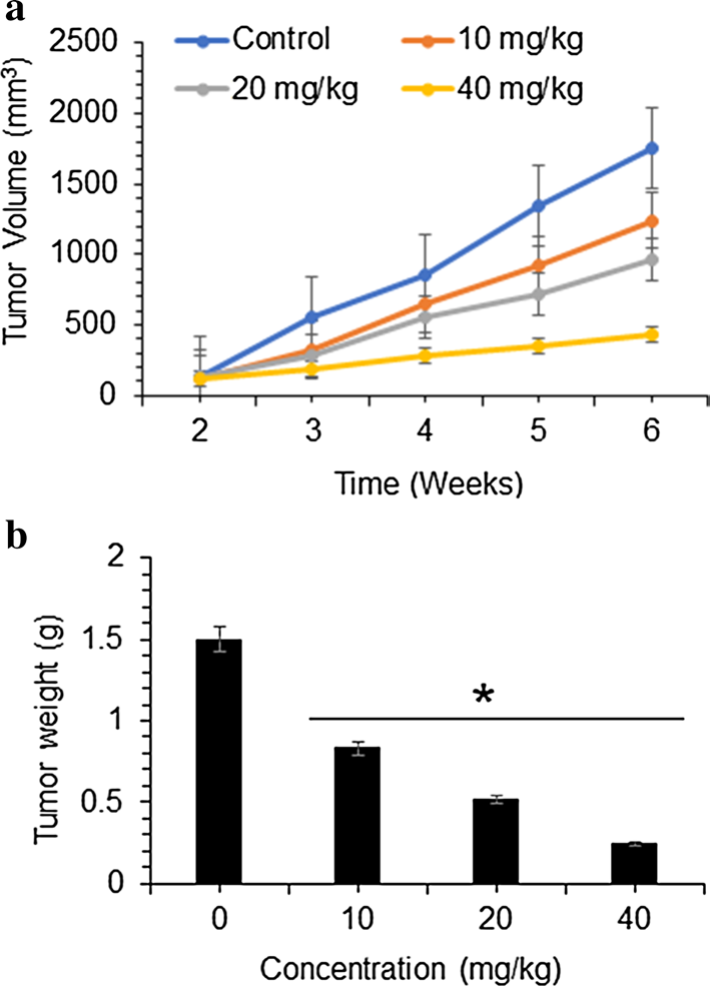
Mukonal inhibits MDA-MB-231 and SK-BR-3 tumor growth in vivo. **a** Tumor volume and **b** tumor weight were measured at indicated time intervals and doses of mukonal molecule. All the experiments for separate drug concentration were executed three times and data was shown as mean ± SE (standard error). The p value of < 0.05 was taken as a statistical significant figure

Nude mice xenografts were used to determine in vivo anticancer effects of mukonal. The results showed that SK-BR-3 tumor growth was remarkably retarded by mukonal administration, in comparison to control group. At the end of 6-weeks, the average tumor volume and weight in untreated control group was substantially advanced than mukonal treated groups. Moreover, the in vivo anticancer effects of mukonal were dose- and time-dependent manner (Fig. 5a, b).

## Discussion

Despite significant advancements in cancer research, management it still remains the most prevalently diagnosed cancer and cause of death in women globally. Better approaches are needed to understand and recognize this disease at molecular levels. Due to timely diagnoses of the disease North America has registered over 80% 5-year survival rate among the breast cancer patients (DeSantis et al. 2016). High frequency of occurrence, late diagnosis and disastrous side effects of present day chemopreventives creates an emergency for novel therapeutic drugs that can deliver better results with higher efficacy (Waks and Winer 2019; Collignon et al. 2016). Mukonal is a carbazole alkaloid and has revealed to possess remarkable pharmacological and biological propensities. It has been reported to induce anticancer effects against different human cancer cell lines (Li et al. 2018; Guo et al. 2019). Therefore, the current study was undertaken due to accumulative evidences suggesting that mukonal has a great propensity to act as an anticancer agent. The results revealed that mukonal decreased the proliferation rate of five variant breast cancer cell lines but remarkable results with IC_50_ of 7.5 µM was recorded against breast cancer MDA-MB-231 and SK-BR-3 cancer cell lines. Mukonal showed a minuscule toxicity against normal breast MB-157 cell line, indicating some specificity of inducing toxicity against cancerous breast cell lines. Mukonal induced potential inhibition of colony generation by MDA-MB-231 and SK-BR-3 cells. Further, chemopreventive drugs target several cellular processes to induce cytotoxicity against cancer cells. Apoptosis has been a focal target of chemopreventives and is often termed as type-I PCD (programmed cell death) (Khursheed et al. 2016). Apoptosis remains dormant in normal cells but in case of injury, malfunction, aging and macromolecule accumulation in cells it plays a vital role of elimination (Bonofiglio et al. 2016). Herein, mukonal induced apoptotic cell death in MDA-MB-231 and SK-BR-3 cell lines in a dose-reliant fashion as suggested by annexin V/FITC assay. Similar results have been reported previously wherein mukonal induced apoptosis in laryngeal cancer cells. Mukonal stimulated apoptosis was further supported by enhanced expression levels of cleaved PARP, cleaved caspase-3 and Bax and retarded expression levels of caspase-3, PARP and Bcl-2 proteins in both the cancerous cell lines after mukonal exposure. Autophagy is another process that remains conserved in multicellular organisms activated under stressful conditions like starvation (Levy et al. 2017). Autophagy is often termed as type-II PCD and also remains as focal target of chemopreventives (Poillet-Perez et al. 2018). Autophagy plays a key role in the degradation of marco-molecules and damaged organelle. This process is completely hallmarked by the formation of autophagosomes, which on maturation turns into autolysosomes (García-Prat et al. 2016). Mukonal was observed to induce autophagic cell death in both MDA-MB-231 and SK-BR-3 cell lines. Similar results have been reported previous wherein mukonal induced autophagic cell death in human nasopharyngeal cancer cells. Both of these cancerous cell lines showed enhanced expressions levels of LC3B-I, LC3B-II and Beclin-1 proteins indicating autophagy initiation at molecular levels.

The in vivo investigation of mukonal revealed that it noticeably retarded growth of MDA-MB-231 and SK-BR-3 breast tumor growth, in comparison to untreated control group and no apparent toxicity was detected. The tumor weight and volume in mice models were observed to decline comparative to increased doses of mukonal.

In conclusion, the results of this investigation revealed that mukonal could potentially induce anti-breast cancer effects both in vitro and in vivo against MDA-MB-231 and SK-BR-3 cell lines. The anti-breast cancer effects of mukonal were observed to mediate through induction of autophagy and apoptosis. These studies point towards the potential of Mukonal in the treatment of breast cancer. However, further in vitro and in vivo studies are required to fully establish it as a lead molecule in the development of breast cancer chemotherapy.

## Data Availability

Not applicable.
